# Bronchial Asthma in Youth: A Brief Concept Review

**DOI:** 10.3390/children12070841

**Published:** 2025-06-26

**Authors:** Roberto W. Dal Negro

**Affiliations:** National Centre for Respiratory Pharmacoeconomics and Pharmacoepidemiology—CESFAR, 37124 Verona, Italy; robertodalnegro@gmail.com

**Keywords:** adolescents’ asthma, children’s asthma, variability of clinical pictures, factors affecting asthma in youth

## Abstract

Bronchial asthma is a respiratory chronic disorder frequently affecting youth. It is characterized by a huge personal, familial, and societal impact. Biological and cellular studies in recent decades define asthma as a chronic inflammatory disease of the airways. Inflammation represents the major pathogenetic factor underlying the airflow obstruction and bronchial hyperactivity that peculiarly characterize asthma. When bronchial asthma is diagnosed after too long a delay and treated too late or inadequately, structural remodeling of the whole bronchial wall can occur and lead to persistent limitations in lung function and quality of life. Although adult asthma and asthma in youth may be recognized by some common pathogenetic mechanisms, there are some important differences that justify a peculiar approach to asthma in young individuals, worth particular attention. Anatomical, physiological, social, and emotional aspects that differentiate asthma in children and adolescence are briefly revised and highlighted in the present review.

## 1. Introduction

Bronchial asthma is the most frequent chronic respiratory disorder occurring in childhood and adolescence that is characterized by an ever-increasing prevalence [1,2].

Although with some geographical differences, asthma tends to improve spontaneously in a great proportion of children all over the world during adolescence even if both morbidity and mortality still remain significant. This intriguing aspect is regarded as mainly related to a missing or a delayed diagnosis of asthma, particularly in adolescents, as they usually tend to underestimate the severity of their asthma symptoms and poorly adhere to the therapeutic regimen prescribed.

However, doctors should be requested to improve their knowledge of asthma, and some crucial medical issues should be pursued more carefully, such as the psychologic aspects of adolescents’ asthma; the proper use of inhalation devices; the need for the continuous and strict monitoring of outcomes; and the assessment of their quality of life. Adolescents are in fact at high risk for medium/long-term negative outcomes because they generally minimize their clinical signs and have substantial difficulties in self-managing.

## 2. Aspects of Natural History

The natural history of asthma is difficult to predict in single individuals. The majority of cases of chronic asthma begin in the first six years of life even if children with asthma can frequently experience a spontaneous complete remission for some years during puberty, and the progression to a severe disease is unusual in all age groups. In general, asthma does not typically affect life expectancy in the absence of other comorbidities and, although fatalities can occur, they are quite infrequent when asthma is properly managed [3].

Several studies on the natural history of asthma over the first six years of life tend to support the hypothesis that there are two groups of children who can complain about asthma-like symptoms early in their life [3,4]. In general, one group consists of subjects showing intermittent asthma symptoms usually related to viral infections. The small size of their airways is regarded as a possible cause of transient wheezing after viral insults in these cases. Respiratory signs tend to disappear progressively as they get older [5,6] in 30–70% of these subjects [7]. The other group consists of subjects who complain about later-onset and persistent respiratory symptoms. They are generally characterized by a personal and family history of atopy or asthma, and the risk for long-persistent asthma later in life is increased in these individuals [7,8].

However, despite the identification of some risk factors, the future evolution of asthma is quite difficult to state individually in real life [9].

Asthma in children and adolescents is recognized by some peculiar aspects. Coughing and wheezing episodes commonly occur in many pediatric diseases, but when properly interpreted, these respiratory signs can contribute to predicting the onset of bronchial asthma and the need for an early therapeutic intervention aimed at limiting future asthma consequences [10].

Unfortunately, warning signs are frequently underestimated in real life, diagnostic procedures skipped, and asthma diagnosis delayed [11].

## 3. Pathogenetic Aspects

Asthma is an inflammatory disease of the airways that involves the whole components of the bronchial wall, such as the epithelium, the muscle, and the vasculature [1]. Bronchial biopsies and bronchoalveolar lavage demonstrated that clear inflammatory features can be found even in patients with very mild forms of newly diagnosed asthma [4,12].

Unfortunately, asthma is diagnosed only after several years of symptoms: note that 30% of young Americans receive their first diagnosis of asthma only at the time of their enlistment [13]. Many doctors still tend to identify asthma in children or adolescents only when the first severe respiratory crisis occurs. In other words, asthma is mostly presumed on a clinical basis in young individuals, with allergic tests and specific lung function measurements being insufficiently requested and/or significantly delayed in real life [14,15,16]. It should be emphasized that not all young individuals may complain about the usual cohort of asthma signs (i.e., wheezing, shortness of breath, prolonged expiration) spontaneously. It was shown that asthma can be revealed only after a methacholine bronchial provocation test in young people complaining about cough as their unique symptom (the so-called “cough variant asthma”) [15]. As this condition is indeed frequent in children and adolescents, these subjects can frequently escape a proper labeling of asthma.

When the proper diagnosis is missing, the appropriate therapeutic strategy is obviously difficult to determine in a timely manner and the progressive impairment of airway structures is unavoidable [17]. Unfortunately, long-term preventive therapies are not sufficiently prescribed, with short-term interventions with symptomatic drugs as needed still being privileged. On the other hand, the conclusion that asthma is a self-limited disease that tends to disappear spontaneously with age is still a very common belief among the majority of patients’ families and also among many doctors.

However, even if many asthmatic children improve substantially during adolescence, this improvement does not occur in all children [17], and functional respiratory limitations can persist up to adulthood in a non-negligible proportion of young subjects [16]. Furthermore, adolescents who suffered from childhood asthma can show persistent airflow imitations and bronchial hyperactivity despite the long-lasting absence of any clinical asthma signs [16]. Finally, children and adolescents only complaining about signs of allergic rhinitis may show bronchial hyperactivity for many years, thus further supporting the hypothesis that their airway tissular structures can remain persistently affected for some decades after the first mild allergic disorders [17].

These assumptions should be shared much more among all health care-givers (namely, pediatricians, general practitioners (GPs), lung physicians) because they strongly support the convenience of an earlier, sustained, and unified interventional strategy oriented to the containment of asthma morbidity (and mortality) in children and adolescents [16].

## 4. Variability of Asthma Presentation by Age

As previously mentioned, the clinical presentation of asthma can frequently change during adolescence by a spontaneous decline in respiratory symptoms. It was shown that around 80% of children who suffered from at least one asthma attack before their first five years of age and around 50% of those who had been classified as asthmatic before this age were asymptomatic when they became teenagers [18,19,20], despite their delayed diagnosis.

Unfortunately, this transient positive evolution frequently leads to the wrong conclusion that asthma has been cured [21,22]. Some factors can contribute to this spontaneous amelioration of asthma: (a) the growth in height that can induce a significant increase in lung volumes and to a spontaneous (though temporary) decrease in bronchial responsiveness; (b) the hormonal changes occurring over the puberty period that can temporarily modulate (particularly in females) the basal immunological responsiveness [21].

A critical and intriguing issue is the sex-dependent evolution of adolescent asthma. In general, the prognosis in females is worse than in males. Actually, females are characterized by a smaller absolute diameter of their airways and by a higher prevalence of allergic phenomena and bronchial hyper-responsiveness during their first decade of life [23]. Unlike during puberty, the incidence of asthma and the severity of symptoms are greater in females after the age of 20 years, presumably due to their stabilized hormonal asset: a condition that is able to affect their airway inflammatory pattern [24]. This peculiar hormonal dependence is further supported by the common evidence of symptom worsening in asthmatic females during their premenstrual and/or menstrual periods that occurs in about one third of cases, which is also characterized by resistance to conventional therapeutic treatments, with high-dose corticosteroids included [25,26]. Likely for these reasons, asthma can complicate pregnancy in youth more often than in adulthood.

## 5. Asthma Prognosis in Youth

The prognosis of asthma appears difficult to evaluate in young individuals. Several studies have documented a tendency towards remission in subjects aged 10–20 years, although the recurrence rate is relatively high even after some years free from symptoms [16,26]. These subjects can still have some degrees of airway obstruction and significant bronchial hyper-responsiveness even in the absence of spontaneous asthma symptoms [26].

Together with female gender, parental atopy, and the onset of wheezing after the first two years of life, residual limitations in lung function, the severity of bronchial hyper-responsiveness, and the clinical severity of asthma during childhood are regarded as the main predicting factors of asthma in adulthood [27,28]. However, asthma is the most significant single cause of disability in the first 18 years of life (i.e., limitations in usual childhood activities; school attendance; sport; sleep; social limitations, family independence) [29]. In other words, adolescents with asthma tend to be continuously critically challenged.

The communication of an asthma diagnosis can elicit intense emotions in parents, with the unpredictable and intermittent course of the disease contributing to determining fears, uncertainties, and incorrect beliefs that also affect asthma management [30].

The psychological profile of adolescents is crucial from this point of view. Adolescents prefer to consider asthma as an episodic condition that only exists during exacerbations and do not accept the therapeutic strategy based on the regular assumption of drugs during asymptomatic periods, albeit aiming to prevent the recurrence of symptoms and avoid the daily limitations in their quality of life [31].

## 6. The Role of Adherence to Treatment

Doctors who have to manage asthmatic adolescents should first be aware of their peculiar poor adherence to regular therapeutic regimens as there is consolidated evidence that around 70% of adolescents usually do not assume their prescribed medications despite the clear written instructions received. This attitude is frequently responsible for the high morbidity of asthma, with near-fatal asthma attacks being 3–6 times more frequent in adolescents compared to older asthmatics [22].

It is known that the adherence to regular treatments is partially influenced by the complexity of the therapeutic regimen. Moreover, inhalation devices are sometimes very complicated to use if the correct procedures for a proper inhalation are not explained. Young asthmatics often use inhalers improperly and pay too poor attention to the procedures needed for effective drug inhalation. In the case of metered dose inhalers (MDIs), the most frequent errors are as follows: they frequently do not shake the canister and do not exhale deeply enough before inhaling; they trigger the spray more than once during a single breath, and they do not hold their breath sufficiently after inhaling. Furthermore, many adolescents perceive inhaler use as an embarrassing condition that reveals their health status limitation that can cause social discrimination [32]. On the other hand, Dry Powder Inhalers (DPIs) also require attention and specific information for their effective utilization as their intrinsic characteristics variably affect their real-life performance [32].

The possible occurrence of side-effects is another factor that frequently limits the adolescents’ willingness to take medications regularly. Moreover, the overestimated parents’ fear that steroids (though inhaled) may affect the children’s or the adolescents’ growth often contribute to their poor adherence, even substantially. It is the doctors’ responsibility to modify this wrong paradigm in these cases, and to inform the patient and their family that the severity of asthma can induce growth reduction *per sé*, while a well-controlled asthma also obtained by inhaled corticosteroids taken for years would favor and allow normal growth [33]. However, in a non-negligible proportion of children, the transient deceleration in growth speed can also be due to the natural delayed onset of puberty and/or to familial genetic factors.

## 7. The Psychological Trait of Young Asthmatics

The personality profile of young asthmatics can be quite fragile [32]. The support of family members is absolutely important for the acceptance and the effective management of asthma in young patients. Conflictual relationships between parents and their asthmatic children/adolescents are more frequent than in control families and are frequently associated with insufficient adherence to regular treatment and then with insufficient asthma control [32]. A family history of chronic affective disorders and substance addiction is not infrequently related to severe or difficult-to-control asthma in young patients. The causal role of the negative parental influence can be suggested by the evidence that asthma symptoms are rapidly and substantially improved during a hospital admission in the majority of these cases [34,35].

Adolescents usually start smoking to feel like adults, or because they are influenced by family models, mass media, and, above all, their friends [35]. Early education aiming to prevent them from starting smoking is crucial and not only motivated to showing direct and systemic tobacco damage, but also to demonstrating that the smoking attitude can favor the development of other dangerous behaviors (i.e., addiction to alcohol, premature sexual activity, and addiction to other substances). It was shown that when adolescents “accept” their asthma and the need to take some respiratory drugs regularly, they skip the majority of these problems in a great proportion of cases [36].

Asthma and psychiatric disorders have been frequently associated in the literature [37], even if the possible underlying causes of this association have not been exhaustively clarified yet. Moderate and severe anxiety, depression, hostility, aggressivity, reduced self-esteem, the need to receive approval from others, and the inability to control emotions are the most frequent personality traits of young asthmatics [32,38]. Compared to non-asthmatic subjects, psychosomatic symptoms (namely, headache and insomnia) are also more common in asthmatic adolescents, presumably also related to asthma-induced forced awakenings during the night. Though still debated, even the role of some respiratory drug might play a role from this point of view.

## 8. Self-Asthma Control

Asthma management requires careful attention to physical signs and the long-term monitoring of clinical outcomes. When instructed, asthmatic adolescents also tend to develop a self-focused cognitive style together to a high sense of personal responsibility for themselves and their future life. Otherwise, asthma management will be negatively affected and emotional/cognitive disorders can become consolidated, thus leading to a growing uncertainty of the future and to inadequate psychological adaptation that can persist for a long time up to their adulthood [39]. The different phases of the relationship between young asthmatics and their asthma are summarized in Table 1.

## 9. The Clinical Approach in Real Life

Accurate medical history is crucial to collect in young asthmatics because it represents the very first step of diagnostic procedures. It should include the following: (1) the type and characteristics of the symptoms and conditions associated with asthma occurrence; (2) the analysis of triggering or aggravating factors; (3) the description of exacerbations; (4) the course of asthma; (5) the knowledge of environmental conditions (both in- and outdoor); (6) the analysis of family history and socio-cultural conditions; (7) the possible existence of comorbidities, with psychological traits included.

For a long time, several clinical elements can suggest or support asthma diagnosis, such as the occurrence of spontaneous wheezing and/or dyspnea especially during the night or at awakening; the sensation of chest tightness; the occurrence of intermittent or chronic cough; the recurrence of “undefined” bronchitis; and respiratory symptoms caused by the exposure to allergens (seasonal or perennial), or by rhinitis or sinusitis, or by physical exercise, particularly in concomitance to atopic manifestations (i.e., conjunctivitis, eczema), and when the familiarity for atopy is known. Nonetheless, all adolescents that report their asthma onset during infanthood should be carefully evaluated [40]. As previously reported, particular attention should be paid to the adolescents’ tendency to minimize their symptoms. In fact, the progressive increase in the caliber of their terminal airways that occurs with body growth can lead to a symptom-free period of various durations that convinces the adolescents (with their families) and some doctors that their asthma has been cured. Unfortunately, they disregard (or do not know) that the underlying tissular conditions supporting asthma (i.e., airway inflammation and bronchial hyperresponsiveness) are still active in asymptomatic or pauci-symptomatic periods of their life.

## 10. Diagnostic Steps

The physical examination and lung function measures are essential steps of the diagnostic asthma pathway. Although not always significant, the physical examination of young asthmatics should nonetheless be completed for supposing or confirming asthma diagnosis. Unavoidable steps are as follows: the inspection of the chest shape and dimension; the inspection of the skin, eyes, and conjunctive status and of the upper respiratory tract; the evaluation of weight, height, nutritional status and stage of maturation, and chest auscultation.

The lung function tests should be performed before and after a short-term bronchodilator (usually salbutamol, 400 mcg). Despite the limited correlation with clinical signs [41], these tests will allow the recognition, qualification, and quantification of the current airflow limitation, even if of subclinical entity. Moreover, the measure of bronchial hyperreactivity to non-specific pharmacological agents and the response to controlled physical exercise represent further useful tools for investigating young patients suspected of asthma, even in the absence of clear respiratory symptoms or of uncertain clinical history [42].

In the majority of young individuals, asthma is associated with sensitization to inhaled allergens and to a family history of atopy. Asthma and bronchial hyper-responsiveness are usually closely linked to serum IgE level and reactivity to skin allergy tests. Therefore, the search for possible sensitizations to most common inhalation allergens (namely, dust mites, animal proteins, pollens, and molds) plays an essential role in the diagnostic pathway for children and adolescents suspected of asthma, particularly during their second decade of life [43]. On the other hand, the long-lasting exposure to these allergens can play a critical role in the pathogenesis of asthma and can cause the persistence and the recurrence of respiratory symptoms.

While frequently under-diagnosed in children and adolescents [44], the school environment can also contribute to revealing asthma in young people thanks to the physical activities usually performed that can work (when properly perceived) as real-life exercise tests in these cases. When asthma has been confirmed according to the complete diagnostic pathway, the prevention of asthma symptoms can start at school by instructing teachers on the importance of pre-treatment with preventer drugs and/or short-term bronchodilators before exercise [8].

When children and adolescents who complain about respiratory symptoms suggestive for atopic asthma are unresponsive to proper asthma therapy, they should be investigated for other etiologies that can mimic the current asthma picture, such as hyperventilation syndrome; psychological problems; mitral valve prolapse (which can be misdiagnosed for exertional asthma due to the chest pain complained about during physical exercise); and vocal cord dysfunction (more frequent in girls with a history of psychological problems or domestic troubles) [45]. Furthermore, the genetic and pharmacological inhibition of cyclooxygenase are further factors that can lead some subjects to experience bronchial obstruction after taking aspirin or NSAIDs. However, a peculiar tendency to develop sinusitis and recurrent nasal polyposis is quite frequent in these cases [46]. The major steps of the clinical and diagnostic asthma approach are summarized in Table 2.

## 11. The Doctor-to-Patient Relationship

In the present period of diminished parental influence, doctors are assuming a crucial role for delivering credible and reliable health information much more than in the past. Peculiar skills are required in doctors who start to manage young asthmatics. In particular, they should adopt an empathetic approach without any paternalistic attitude, while active listening techniques are crucial for favoring the collection of the adolescent’s story, with psychological traits included [46,47,48,49]. The adolescent should feel free to ask any question regarding their asthma, while the doctor should make sure that they have understood all information concerning the major risk factors; the need for asthma prevention; how and when respiratory drugs should be taken; the crucial role of the adherence to the therapeutic strategy prescribed; how to manage the inhalation devices effectively; the need for the periodical assessment of lung function; what the adolescent can expect for their future life; and when a further doctor visit would be really needed. On the other hand, a poor doctor-to-patient relationship will affect the effectiveness of asthma management dramatically with the consequent drop in short-term and long-term clinical outcomes and in adolescents’ quality of life. In other words, the role of education provided by doctors or by expert care-givers is irreplaceable [32,48,49,50].

Further to the direct doctor’s role, several specific apps and electronic tools are presently available for favoring asthma knowledge and asthma self-management. As it is well known how much adolescents enjoy using technology more than written documents, these tools can also be advantageous from this point of view, though the relationship is much more impersonal.

Another interesting approach for supporting a proper asthma acceptance in young people is their participation in self-help groups where other subjects of the same age who had already experienced asthma can share their personal experience and beliefs with the new member. The language is quite open in these cases and the majority of psychological barriers disappear, thus creating a favorable environment for the novel adolescent included. Furthermore, a proper educational program should also be delivered to the children’s and the adolescents’ parents with the aim to further contribute to optimizing a global anti-asthma strategy in favor of their sons and daughters. Finally, since asthma is a very common and ever-increasing condition among young people, it would be desirable to deliver a general short training on asthma to school teachers and sport coaches with the aim to improve their basic knowledge of major asthma risk factors, of actions to be avoided, and of actions to take in emergency conditions. Specific pamphlets and cartoons could be very helpful from this point of view.

## 12. Conclusions

The main purpose of all the actions and activities mentioned above is to make young asthmatics (child and adolescent) not feel limited in their life and equally perform when compared to non-asthmatic subjects of the same age.

The vast majority of cases of asthma in youth are not and do not become a disabling condition if diagnosed early, comprehensively approached, and properly managed. As proof, several young Olympic and World Champion medalists are asthmatic.

## Figures and Tables

**Table 1:** Different phases of young asthmatics’ relationship with their own asthma.

*1—The negative phase*	
	Initial perception of asthma symptoms; Fear of asthma symptoms (after confirmation by expert care-givers); Fear of chronic condition and related limitations; Resigned acceptance of asthma; Renunciation of asthma control; Disregard of treatment options; Social isolation.
*2—The positive phase*	
	Start of reasoning process; Active search for asthma knowledge; Awareness and acceptance of asthma chronic condition; Operational options considered; Decision to become the owner of their chronic condition; Start of self-monitoring; Optimization of social and familial relationships.

**Table 2:** The major steps of the clinical and diagnostic approach to asthma.

○ Step 1 ▪ Symptom collection; ▪ Check of familial history/socio-economic conditions; ▪ Check of psychological traits; ▪ Check of triggers (environment and smoke, including the use of other substances); ▪ Check of comorbidities.
○ Step 2 ▪ Physical examination and lung function measures; ▪ Diagnosis confirmation; ▪ Minimize risk factors; ▪ Drug prescriptions; ▪ Non-pharmacological strategies (when needed); ▪ Educational approach to inhalation devices.
○ Step 3 ▪ Check of short- and long-term outcomes; ▪ Check of drugs’ side-effects; ▪ Check of adherence to treat.
